# Vitamin B12 and Folate Status in Pregnant Females and Their Infants in Norway: Secondary Analysis from the Mommy’s Food Study

**DOI:** 10.1016/j.tjnut.2023.10.013

**Published:** 2023-10-18

**Authors:** Sol Maja G Bjørkevoll, Carolien Konijnenberg, Ingrid Kvestad, Adrian McCann, Per M. Ueland, Synnøve Næss Sleire, Lisbeth Dahl, Marian Kjellevold, Tor A. Strand, Maria W. Markhus

**Affiliations:** 1 Innlandet Hospital Trust, Lillehammer, Norway; 2 Centre for International Health, University of Bergen, Norway; 3 Department of Psychology, Inland Norway University of Applied Sciences, Lillehammer, Norway; 4 Regional Centre for child and Youth Mental Health and Child Welfare, NORCE, Bergen, Norway; 5 Bevital AS, Bergen, Norway; 6 Institute of Marine Research (IMR), Bergen, Norway

**Keywords:** infants, pregnancy, breastfeeding, cobalamin, folate, homocysteine, methylmalonic acid, cB12, Mommy’s Food Study, supplement, micronutrient status

## Abstract

**Background:**

Vitamin B12 and folate are essential micronutrients important for normal infant growth and development.

**Objectives:**

The aims were to describe vitamin B12 and folate status in pregnant females and their infants according to commonly used status cutoffs and examine the associations between maternal status, maternal supplement use, and breastfeeding and infant status.

**Methods:**

Pregnant females were recruited at 18 wk gestation in Bergen, Norway. Maternal vitamin B12 and folate status were measured at gestational weeks 18 (*n* = 136) and 36 (*n* = 116), and infant status was measured at ages 3 (*n* = 73) and 6 (*n* = 74) mo.

**Results:**

At gestational weeks 18 and 36, respectively, 4.4% and 2.6% of the mothers had plasma cobalamin concentrations <148 pmol/L, 0.7% and 6.9% had methylmalonic acid (MMA) concentrations >0.26 μmol/L, and 3.7% and 30% had folate concentrations <10 nmol/L. None of the females had total homocysteine (t-Hcy) concentrations >13 μmol/L or 3 combined indicator of vitamin B12 (cB12) < −0.5. At 3 and 6 mo, respectively, 4.1% and 5.4% of the infants had cobalamin concentrations <148 pmol/L, 63% and 74% had t-Hcy concentrations >6.5 μmol/L, 59% and 66% had MMA concentrations >0.26 μmol/L, and 47% and 60% had cB12 > −0.5. None of the infants had folate concentrations <10 nmol/L. Several of the vitamin B12 biomarkers in infants were associated with maternal vitamin B12 status during pregnancy. Breastfed infants had lower vitamin B12 status (as indicated by plasma cobalamin, t-Hcy, and cB12) than nonbreastfed infants at both 3 and 6 mo. Use of supplements during pregnancy was associated with better vitamin B12 status among infants at 3 and 6 mo, as indicated by infants’ cobalamin and t-Hcy concentrations.

**Conclusions:**

Subclinical vitamin B12 deficiency among infants was common and associated with maternal vitamin B12 status during pregnancy and breastfeeding. Among the mothers, an increase in biochemical folate deficiency was discovered toward the end of gestation. Further studies are needed to investigate clinical consequences.

This trial was registered at clinicaltrials.gov as NCT02610959.

## Introduction

The micronutrients vitamin B12 (cobalamin) and folate are closely related metabolically and involved in several processes in the body, including maturation and division of cells and myelination of the nervous system [1,2]. These vitamins are therefore essential for normal growth and development of the fetus during pregnancy and in the first years of life [2,3]. Pregnant females and infants are recognized to be at particular risk of vitamin B12 deficiency [4]. Studies from high- and low-income settings around the world have reported high prevalences of biochemical signs of subclinical vitamin B12 deficiency in infants [[5], [6], [7], [8], [9], [10]]. Furthermore, a recent meta-analysis including 57 studies worldwide estimated that 25% of pregnant females had poor vitamin B12 status during gestation [11]. The importance of folate in pregnancy is well recognized, and fortification programs and supplementation of folate before and during early pregnancy are implemented globally [12,13]. In Norway, it is advised to take a daily folate supplement of 400 μg from preconception through the first 12 wk of pregnancy [14].

Despite the reports of the high prevalence of poor vitamin B12 status among infants, overt and subclinical deficiency may be difficult to detect. Signs and symptoms of vitamin B12 deficiency in infants are often diffuse and overlap with other conditions. Symptoms may include failure to thrive, irritability, refusal of solid foods, megaloblastic anemia, and developmental regression [15]. Furthermore, subclinical vitamin B12 status may be asymptomatic. Therefore, the diagnosis of deficiency and subclinical vitamin B12 status is often dependent on biochemical testing.

Several biochemical indicators of vitamin B12 and folate status are available. Total cobalamin is a widely used direct measure reflecting total circulating vitamin B12 status [16]. Methylmalonic acid (MMA) is a product of a reaction that requires cobalamin as a cofactor, where circulating MMA concentrations rise with poor vitamin B12 status [17]. When assessing vitamin B12 status, it is recommended to use more than one biomarker [18], reflected by the development of the combined indicator of vitamin B12 (cB12) status developed by Fedosov et al. [19]. cB12 includes 2 to 4 biomarkers of vitamin B12 accounting for folate status and age when estimating vitamin B12 status [16,19]. Conversion of homocysteine (Hcy) in 1-carbon metabolism is dependent on both vitamin B12 and folate. Therefore, total Hcy (t-Hcy) is used as a functional biomarker of both vitamin B12 and folate status [17], where deficiency or low status of either of these vitamins may lead to elevated t-Hcy concentrations [16,17]. In adults, t-Hcy is primarily considered an indicator of folate status [20], while in infants, there is a consensus that t-Hcy is the preferred indicator of vitamin B12 status [16]. In addition, folate may be measured using serum/plasma folate concentrations, which is widely used for evaluating folate status [21].

Little is known about vitamin B12 and folate status in Norwegian pregnant females and their infants. Therefore, we aimed to describe vitamin B12 and folate status among pregnant females and their infants in Norway using indicators of vitamin B12 (plasma cobalamin, t-Hcy, MMA, and cB12) and folate status (plasma folate and t-Hcy). We also examined the relationship between maternal status in pregnancy and infant status during their first 6 mo of life and investigated whether maternal dietary supplement use during pregnancy and breastfeeding affected infants’ vitamin B12 or folate status.

## Methods

### Study design and participants

**FIGURE 1:**
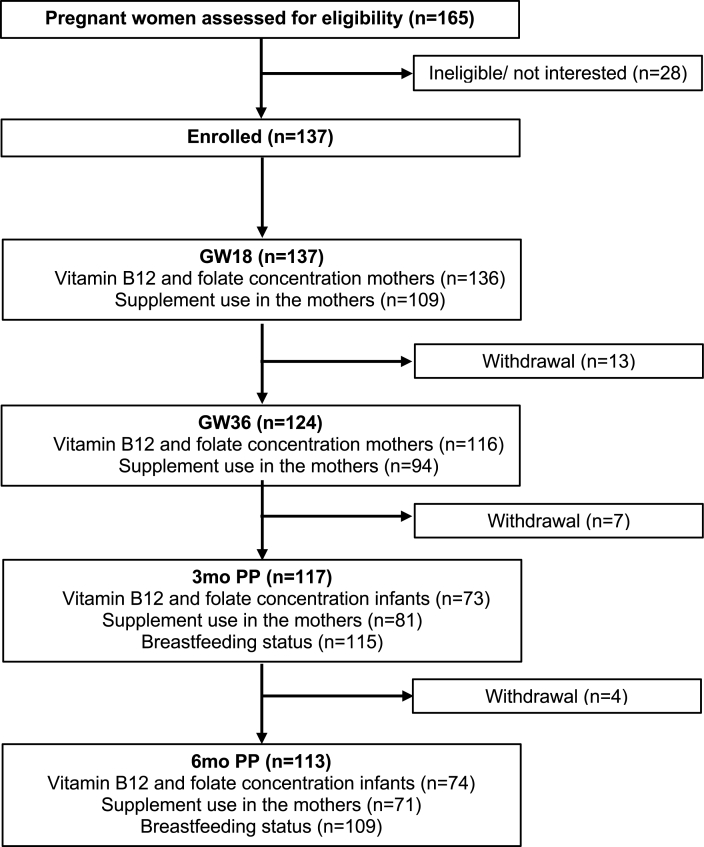
Flowchart of the study population. Abbreviations: GW18, gestation week 18; GW36, gestation week 36; PP, postpartum.

The present study is a secondary analysis from the “Mommy’s Food” study (NCT02610959), which was conducted in Bergen, Norway (2016–2018). The study was a randomized controlled trial, where a total of 137 pregnant females were assigned to either receive Atlantic cod (*Gadus morhua*) twice weekly or to continue with their habitual diet from gestational week (GW) 20 to 36. The main outcome of the study was to investigate the difference in urinary iodine concentrations between groups before and after the intervention. In this secondary analysis, the pregnant females and infants from both study arms were included in an observational cohort design. Further information about the study design and protocol is described in detail elsewhere [22,23]. Pregnant females were recruited from January 2016 until February 2017 prior to their routine ultrasound at GW 18 at the Women’s Health Clinic at Haukeland University Hospital, Bergen, Norway. Recruitment through online broadcasts was also used to achieve a higher participation rate. The pregnant females were followed-up, and data were collected at GW 18 and 36 and further with their infants at 3 and 6 mo of age. A flow chart of the study population, the number of participants, and the available data collected is given in Figure 1.

The inclusion criteria included pregnant females with primiparous singleton pregnancies in GW ≤19 who spoke and/or understood Norwegian writing. Given the intervention with Atlantic cod, females who were allergic to fish or had chronic diseases affecting iodine status were excluded. All study participants provided written informed consent. The trial was conducted in accordance with the Declaration of Helsinki and was approved by the Regional Ethical Committee for Medical and Health Research Ethics West (2015/897).

### Blood sampling

Blood samples were collected from the pregnant females at GW 18 and 36 and from the infants at ages 3 and 6 mo. Venous blood for plasma preparation was collected in BD Vacutainer K2EDTA 5.4 mg (adults) and 3.6 mg (infants) vials and centrifuged (1000–1300 × *g*, 20°C, 10 min) within 30 min prior to plasma separation. Capillary blood samples from the infants’ heel or fingertip were collected if venipuncture was not possible. The fingertip or heel was warmed with a hot water balloon prior to the capillary blood sampling to ensure sufficient blood flow. For the heel pricks, a Tenderfoot ITC heel-incision device (Accriva Diagnostics) was used. For the finger pricks, ACCU-Check Safe-T-Pro Plus lancets (Roche Diagnostics) was used. After collection, the capillary blood was placed in a BD Microtainer Blood Collection Tube K2EDTA (Becton, Dickinson and Co.) and centrifuged (1000–1300 × *g*, 20°C, 10 min) within 30 min prior to plasma separation. The infants were given 1 to 2 mL of 25% sucrose-water solution for pain relief prior to the sampling.

### Analyses of vitamin B12 and folate status

Plasma samples were analyzed at Bevital Laboratory, Bergen, Norway (www.bevital.no). Concentrations of plasma cobalamin and folate were analyzed by microbiological assays based on colistin sulfate-resistant strain of *Lactobacillus leichmannii* [24] and a chloramphenicol-resistant strain of *Lactobacillus casei* [25], respectively. The within-day coefficient of variation (CV) for cobalamin and folate was 4%, while the between-day variation was 5% for both biomarkers [26]. Gas chromatography-tandem mass spectrometry based on methyl chloroformate derivatization was used to determine plasma t-Hcy and MMA concentrations [26]. The within-day CV ranged from 1% to 5%, whereas the between-day CV ranged from 1% to 3%.

### Definition of vitamin B12 and folate status

Plasma cobalamin, t-Hcy, and MMA concentrations were used to describe vitamin B12 status. cB12 status was calculated as the log of (cobalamin/t-Hcy×MMA) multiplied by a constant while adjusting for folate concentration and age [19]. Higher cB12 indicates better vitamin B12 status. Plasma folate and t-Hcy concentrations were used to assess folate status. An overview of the cutoffs used to define low concentrations of vitamin B12 and folate is provided in Table 1 [27, 28].

**TABLE 1:** Cutoffs used to assess vitamin B12 and folate status

Indicator	Cutoff	Definition
Vitamin B12 status		
Cobalamin	<148 pmol/L	Low B12-status [20,27]
t-Hcy	>13 μmol/L (mothers)	Elevated t-Hcy [16]
	>6.5 μmol/L (infants)	Subclinical B12 deficiency [16]
MMA	>0.26	
cB12	< −0.5	Low B12 status [19]
Folate status		
Folate	<10 nmol/L	Folate deficiency [28]
t-Hcy	>13 μmol/L (mothers)	Elevated t-Hcy [16]

Abbreviations: cB12, combined indicator for vitamin B12 status based on the concentrations of t-Hcy, MMA, and cobalamin; MMA, methylmalonic acid; t-Hcy, total homocysteine.

### Breastfeeding status

Information regarding breastfeeding status was collected using 24-h dietary recalls conducted during the study visits at 3 and 6 mo postpartum. In the statistical analyses, the breastfeeding status was categorized as: *1*) exclusively and partly breastfed infants or *2*) nonbreastfed infants. Exclusively breastfed was defined using WHO’s definition of exclusively breastfeeding: “breastfeeding with no other food or drink, not even water.” With this definition, prescribed medication, vitamins, and minerals are not counted as fluids or foods [29].

### Maternal vitamin B12 supplement use

Information regarding maternal supplement use was collected from a food frequency questionnaire (FFQ) completed at GW 18 and 36 and 3 and 6 mo postpartum. The FFQ was electronic and semiquantitative and aimed to capture participants’ habitual diet and dietary supplement use [30]. In the FFQ completed in GW 18, participants were asked to report an estimate of their diet since they became pregnant, whereas the FFQ in GW 36 covered the intake during the last 16 wk (approximately since the last completed FFQ). The FFQs completed at 3 and 6 mo postpartum were intended to cover participants’ diet during the past 3 mo. The questions regarding dietary supplement use included type and intake frequency. In the statistical analyses, participants were categorized into 2 categories: *1*) B-vitamin supplement users: females that took a multivitamin and/or a supplement with B vitamins or *2*) non-B vitamin supplement users: those only taking other supplements (not B vitamins or multivitamins) or no supplements.

### Statistical analysis

For the baseline characteristics, categorical variables are reported as frequencies and percentages, while continuous variables are reported as mean values (SD) for normally distributed variables, and medians and interquartile ranges (IQRs) for variables with a skewed distribution. The correlations between the different biomarkers and between different time points were estimated using the Spearman rank order correlation. The associations were considered weak, moderate, or strong if the correlation coefficient (rho) was >0.10, >0.40, or >0.70, respectively [31]. Dose-response curves displaying the associations between maternal (GW 18 and 36) and infant (3 and 6 mo) status cB12 and folate status were also made using the “twoway fpfitci” command in STATA. Generalized linear models with an identity link function and of the Gaussian distribution family were used to investigate the association between maternal dietary supplement use and breastfeeding with infant biomarker-concentrations at ages 3 and 6 mo. The outcome variables (cobalamin, t-Hcy, MMA, and folate) were log-transformed to approximate normal distribution. Statistical analyses were performed using STATA version 17 (StataCorp). *P* values < 0.05 were considered statistically significant.

## Results

A total of 137 of the 165 eligible pregnant females agreed to participate in the study (Figure 1). The pregnant females who were included in the study had an average age of 29 y. Most were either cohabiting or married and had a medium to high household income. At 3 mo of age, 79% of the infants were exclusively breastfed, while at 6 mo of age, only 3% was exclusively breastfed, with the majority (90%) of infants being partially breastfed. Data on supplement use in the infants were collected from 115 participants at 3 mo and 109 participants at 6 mo of age. Among these infants, 3 were found to be using supplements containing folate and other B vitamins at 3 mo, while only 1 infant was using such supplement at 6 mo of age. None of the infants reported the use of supplements containing vitamin B12 at either 3 or 6 mo of age. Demographic information and baseline characteristics of the mothers and infants included in the study are summarized in Table 2.

**TABLE 2:** Baseline characteristics of mothers and infants in the Mommy’s Food Study

Characteristics	*n*	mean (SD) or %
Mothers		
Age, y	135	29.3 (3.8)
BMI before pregnancy^1^, kg/m^2^	132	23.1 (4.0)
BMI during pregnancy^2^, kg/m^2^	118	24.5 (4.0)
Education, y		
≤13	19	14.3
14–17	33	24.8
>17	81	60.9
Household income, NOK		
Low (<549,999)	39	29.3
Medium (550,000–1,249,999)	77	57.9
High (>1,250,000)	17	12.8
Marital status		
Single or divorced	2	1.5
Cohabitant	85	63.9
Married	43	32.3
Other	3	2.3
Supplement users^3^		
GW18		
Users	79	72.5
Nonusers	30	27.5
GW36		
Users	53	43.6
Nonusers	41	56.4
3 mo postpartum		
Users	39	48.2
Nonusers	42	51.9
6 mo postpartum		
Users	29	40.9
Nonusers	42	59.2
Infants		
Sex (female)	58	50.4
Birth weight, g	93	3491 (536)
GA at time of delivery, wk	122	40.2 (2.1)
Preterm delivery^4^	6	4.9
Breastfeeding status, 3 mo		
Not breastfed	5	4.4
Partially breastfed	19	16.5
Exclusively breastfed	91	79.1
Breastfeeding status, 6 mo		
Not breastfed	8	7.3
Partially breastfed	98	89.9
Exclusively breastfed	3	2.8

Abbreviations: BMI, body mass index; GA, gestational age; GW18, gestation week 18; GW36, gestation week 36; NOK, Norwegian Krone; SD, standard deviation.

Values are presented as means (SDs) or percentages.

1 Calculated using self-reported height and previous weight.

2 Calculated using self-reported current weight at gestation week 18 of the pregnancy.

3 Users defined as those taking B-vitamins and/or multivitamins, nonusers defined as those only taking other types of supplements or no supplement.

4 Preterm delivery defined if child was born before gestation week 37.

Direct and functional measures of vitamin B12 and folate status for the pregnant females and infants are presented in Table 3, together with the distribution of the participants according to the different biomarker cutoffs. At GW 18 and 36, 4.4% and 2.6% of the females had cobalamin concentrations <148 pmol/L, while 0.7% and 6.9% of the females had MMA concentrations >0.26 μmol/L, respectively. None of the mothers had t-Hcy >13 μmol/L or cB12 < −0.5 during pregnancy, indicating adequate vitamin B12 status. At GW 18, 3.7% of the pregnant females had folate concentrations <10 nmol/L, increasing to 30.2% at GW 36, indicating decreasing folate status throughout gestation. A total of 4.1% and 5.4% of the infants had cobalamin concentrations <148 pmol/L at 3 and 6 mo of age, respectively. At 3 mo of age, 63.0%, 58.9%, and 46.6% of the infants had t-Hcy >6.5 μmol/L, MMA >0.26 μmol/L and cB12 < −0.5, respectively, suggesting subclinical vitamin B12 deficiency. At 6 mo of age, the proportions increased to 74.3%, 66.2%, and 59.5%, indicating decreasing vitamin B12 status among the infants during the first 6 mo of life. None of the infants had folate concentrations <10 nmol/L at either 3 or 6 mo of age, indicating sufficient folate status.

**TABLE 3:** Vitamin B12 and folate status in the mothers and infants

	Mothers				Infants			
	GW18 (*n* = 136)^1^		GW36 (*n* = 116)^2^		3 mo (*n* = 73)^3^		6 mo (*n* = 74)	
	Median (IQR)	Def, *n* (%)	Median (IQR)	Def, *n* (%)	Median (IQR)	Def, *n* (%)	Median (IQR)	Def, *n* (%)
Cob	220.0 (184.5, 301.9)	6 (4.4)	224.4 (187.9, 291.1)	3 (2.6)	244.9 (199.0, 321.8)	3 (4.1)	221.6, (194.2, 324.8)	4 (5.4)
t-Hcy	4.7 (4.3, 5.3)	0 (0.0)	5.7 (4.9, 6.5)	0 (0.0)	7.3 (6.2, 8.8)	46 (63.0)	7.5 (6.5, 8.8)	55 (74.3)
MMA	0.1 (0.1, 0.2)	1 (0.7)	0.2 (0.1, 0.2)	8 (6.9)	0.4 (0.2, 0.9)	43 (58.9)	0.5 (0.2, 1.0)	49 (66.2)
Folate	26.4 (16.4, 34.9)	5 (3.7)	14.2 (9.4, 27.3)	35 (30.2)	42.0 (34.7, 58.7)	0 (0.0)	45.7 (34.2, 63.4)	0 (0.0)
cB12	0.7 (0.5, 0.9)	0 (0.0)	0.4 (0.2, 0.7)	0 (0.0)	−0.4 (−1.0, 0.2)	34 (46.6)	−0.7 (−1.0, 0.0)	44(59.5)

Abbreviations: cB12, combined indicator for vitamin B12 status based on the concentrations of t-Hcy, MMA, and cobalamin; Cob, cobalamin, Def, deficiency; GW18, gestation week 18; GW36, gestation week 36; IQR, interquartile range; MMA, methylmalonic acid; t-Hcy, total homocysteine.

Values are presented at medians and IQRs. The following cutoff values were used for calculating the percentage with low status of the biomarkers: Cob: <148 pmol/L; t-Hcy: >13 μmol/L for the mothers and >6.5 μmol/L for the infants; MMA: >0.26 μmol/L; folate: <10 nmol/L; and cB12: <−0.5.

1 *n* = 134 for cB12 at GW18.

2 *n* = 114 for cB12 and cobalamin at GW36.

3 *n* = 72 for folate concentrations in infants 3 mo.

**FIGURE 2:**
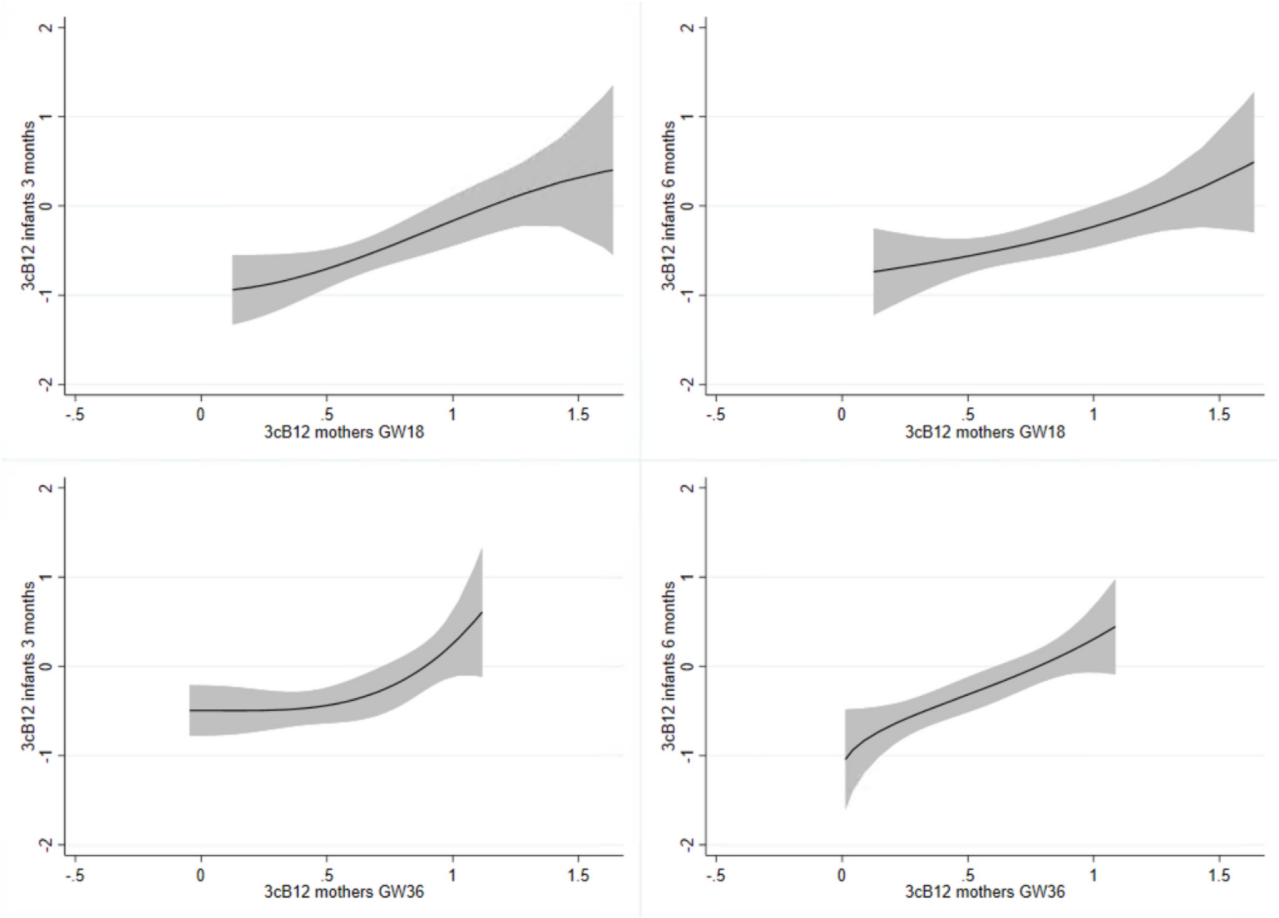
Dose-response curves of cB12 concentrations in the mothers and infants. The figure displays the relationship between mothers’ cB12 status during pregnancy and infants’ cB12 status at 3 and 6 mo of age with 95% confidence intervals. Abbreviations: cB12, combined indicator for vitamin B12 status based on the concentrations of total homocysteine, methylmalonic acid, and cobalamin; GW18, gestation week 18, GW36, gestation week 36.

**FIGURE 3:**
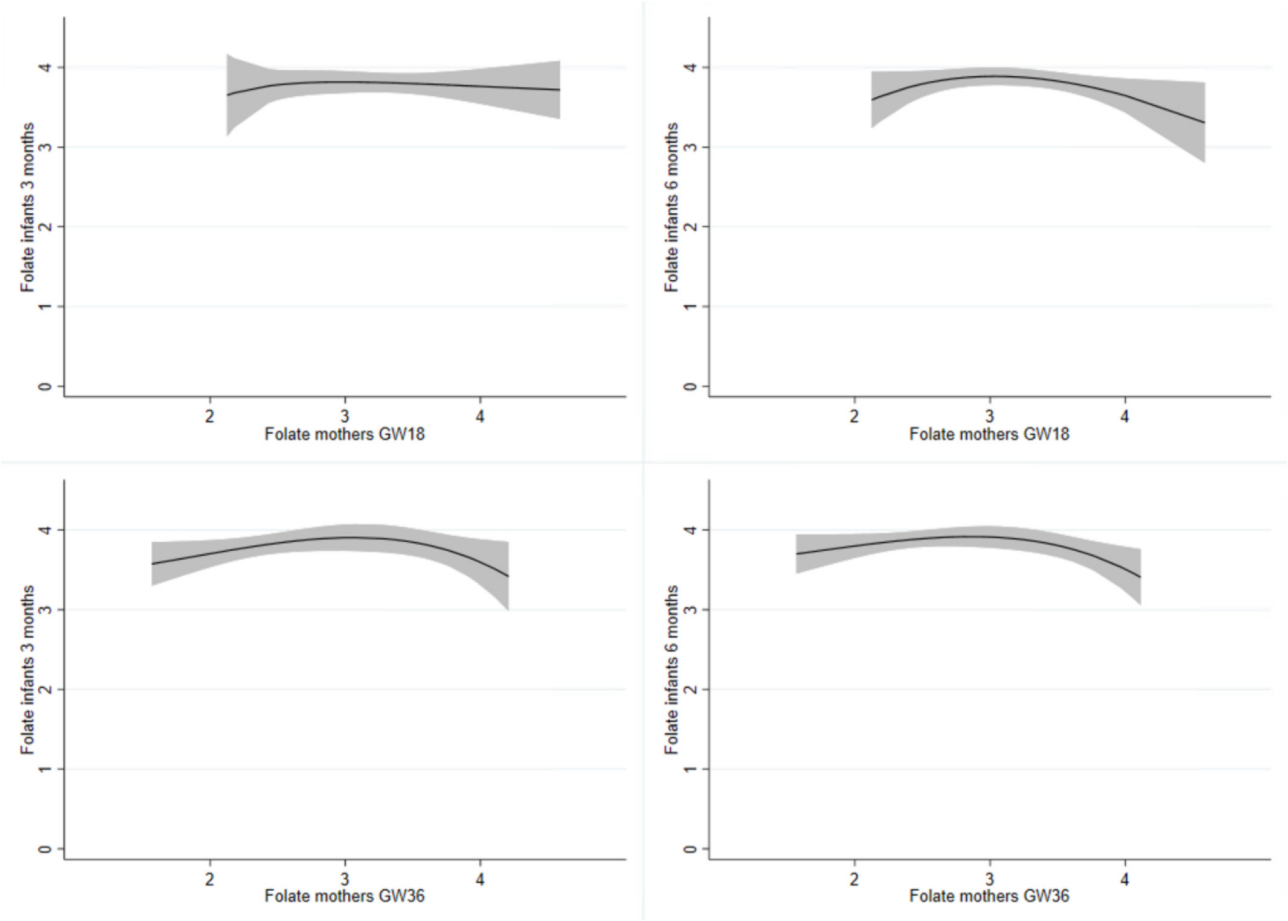
Dose-response curves of log-transformed folate concentrations in the mothers and infants. The figure displays the relationship between mothers’ folate status during pregnancy and infants’ folate status at 3 and 6 mo of age with 95% confidence intervals. Abbreviations: GW18, gestation week 18; GW36, gestation week 36.

Several of the indicators for vitamin B12 status indicators in the infants (cobalamin, t-Hcy, and cB12) were associated with indicators of vitamin B12 status in the mothers (cobalamin, MMA, t-Hcy, and cB12) during pregnancy (Table 4). As depicted in Figure 2, maternal cB12 status during pregnancy at GW18 and GW36 was moderately associated with infant cB12 status at age 3 and 6 mo (*P* < 0.001). Maternal folate status had a weak association with infants’ t-Hcy status at 3 mo and infant cobalamin status at 6 mo but not with infant folate status at any of the time points (Table 4 and Figure 3).

**TABLE 4:** Correlation matrix of vitamin B12 and folate status in the mothers and infants

		Mothers GW18 (*n* = 136)					Mothers GW36 (*n* = 116)				
		Cobalamin	t-Hcy	MMA	Folate	cB12	Cobalamin	t-Hcy	MMA	Folate	cB12
Infants 3 mo (*n* = 73)	Cobalamin	0.36 (0.002)	−0.29 (0.013)	−0.15 (0.218)	0.21 (0.075)	0.37 (0.001)	0.39 (0.001)	−0.25 (0.037)	−0.27 (0.022)	0.25 (0.036)	0.44 (<0.001)
	t-Hcy	−0.28 (0.017)	0.18 (0.124)	0.21 (0.081)	−0.27 (0.023)	−0.33 (0.004)	−0.39 (0.001)	0.40 (<0.001)	0.34 (0.004)	−0.32 (0.006)	−0.54 (<0.001)
	MMA	−0.15 (0.196)	0.06 (0.598)	0.32 (0.006)	0.04 (0.730)	−0.29 (0.013)	−0.22 (0.077)	−0.02 (0.884)	0.31 (0.010)	0.08 (0.521)	−0.26 (0.031)
	Folate	0.14 (0.265)	0.01 (0.909)	−0.01 (0.965)	−0.06 (0.627)	0.03 (0.831)	0.06 (0.655)	−0.10 (0.405)	−0.05 (0.685)	0.01 (0.904)	0.08 (0.534)
	cB12	0.26 (0.026)	−0.19 (0.113)	−0.34 (0.004)	0.04 (0.735)	0.41 (<0.001)	0.33 (0.006)	−0.14 (0.243)	−0.38 (0.001)	0.04 (0.732)	0.43 (<0.001)
Infants 6 mo (*n* = 74)	Cobalamin	0.22 (0.060)	−0.15 (0.217)	−0.07 (0.578)	0.24 (0.043)	0.22 (0.059)	0.29 (0.014)	−0.19 (0.119)	−0.17 (0.142)	0.29 (0.016)	0.31 (0.009)
	t-Hcy	−0.13 (0.287)	0.16 (0.184)	0.25 (0.031)	−0.18 (0.126)	−0.24 (0.036)	−0.21 (0.076)	0.27 (0.021)	0.36 (0.002)	−0.13 (0.287)	−0.42 (<0.001)
	MMA	−0.17 (0.146)	0.07 (0.528)	0.43 (<0.001)	0.05 (0.700)	−0.40 (<0.001)	−0.27 (0.025)	0.19 (0.106)	0.48 (<0.001)	0.01 (0.923)	−0.49 (<0.001)
	Folate	0.18 (0.130)	−0.04 (0.726)	−0.04 (0.737)	−0.06 (0.612)	0.10 (0.397)	0.07 (0.580)	0.06 (0.618)	−0.09 (0.469)	−0.07 (0.575)	0.04 (0.767)
	cB12	0.23 (0.053)	−0.09 (0.442)	−0.42 (<0.001)	0.06 (0.612)	0.43 (<0.001)	0.36 (0.002)	−0.26 (0.031)	−0.52 (<0.001)	0.11 (0.357)	0.59 (<0.001)

Abbreviations: cB12, combined indicator for vitamin B12 status based on the concentrations of t-Hcy, MMA, and cobalamin; GW18, gestation week 18; GW36, gestation week 36; MMA, methylmalonic acid; t-Hcy, total homocysteine.

Pairwise Spearman correlation was used investigate the relationship between the infants and mothers’ biomarkers for folate and vitamin B12 status. The results are presented as Spearman’s rho (*P* value). Significant values in bold.

Breastfed infants had lower vitamin B12 status, as indicated by plasma cobalamin and t-Hcy concentrations, and cB12, compared with nonbreastfed infants at 3 and 6 mo of age (Table 5 and Table 6).

**TABLE 5:** Infants’ vitamin B12 and folate status according to breastfeeding status (*n* = 73^1^)^2^

	Log cob		Log t-Hcy		Log MMA		Log folate		cB12	
	Exp (95%CI)	*P*	Exp (95%CI)	*P*	Exp (95%CI)	*P*	Exp (95%CI)	*P*	Coef. (95%CI)	*P*
Infant status 3 mo										
BF status 3 mo										0.041
Non-BF (*n* = 3)	Ref		Ref		Ref		Ref		Ref	
BF (*n* = 70)	0.64 (0.43, 0.94)	0.023	1.44(1.04, 2.00)	0.030	2.04(0.60, 6.94)	0.251	0.63(0.38, 1.06)	0.080	−0.94(−1.83, −0.04)	0.041
Infant status 6 mo										
BF status 6 mo										
Non-BF (*n* = 7)	Ref		Ref		Ref		Ref		Ref	
BF (*n* = 66)	0.50 (0.40, 0.64)	<0.001	1.55 (1.30, 1.85)	<0.001	3.15 (1.58, 6.29)	0.001	0.70 (0.52, 0.93)	0.015	−1.40 (−1.89, −0.90)	<0.001

Abbreviations: BF, breastfed; cB12, combined indicator for vitamin B12 status based on the concentrations of t-Hcy, MMA, and cobalamin; CI, confidence interval; cob, cobalamin; GW18, gestation week 18, GW36, gestation week 36; MMA, methylmalonic acid; PP, postpartum; Ref, reference; t-Hcy, total homocysteine.

Generalized linear models were used to investigate the infant’s status at 3 and 6 mo depending on the breastfeeding status at 3 and 6 mo. Significant values in bold.

1 *n* = 72 for Log folate 3 mo.

2 The results are presented as Exp (exponentiated coefficients and corresponding 95% confidence intervals) or Coef (coefficient estimates and corresponding 95% confidence intervals).

**TABLE 6:** Vitamin B12 status in the infants according to breastfeeding status

	3 mo				6 mo			
	BF(*n* = 70)		Non-BF (*n* = 3)		BF (*n* = 66)		Non-BF (*n* = 7)	
	Median (IQR)	Def, *n* (%)	Median (IQR)	Def, *n* (%)	Median (IQR)	Def, *n* (%)	Median (IQR)	Def, *n* (%)
Cob	239.1 (194.9, 313.0)	3 (4.3)	390.8 (364,0, 446.8)	0 (0.0)	212.8 (188.2, 277.1)	4 (6.1)	461.0, 374,5, 562,4)	0 (0.0)
t-Hcy	7.5 (6.3, 9.0)	46 (65.7)	4.7 (4.7, 6.1)	0 (0.0)	7.8 (6.7, 9.0)	54 (81.8)	5.6 (4.3, 5.6)	0 (0.0)
MMA	0.4 (0.2, 1.0)	42 (60.0)	0.2 (0.2, 0.3)	1 (33.3)	0.6 (0.3, 1.1)	49 (74.2)	0.2 (0.2, 0.3)	0 (0.0)
cB12	−0.5 (−1.0, 1.4)	34 (48.6)	0.5 (0.3, 0.5)	0 (0.0)	−0.8 (−1.1, −0.2)	44 (66.7)	0.6 (0.6, 1.1)	0 (0.0)

Abbreviations: cB12, combined indicator for vitamin B12 status based on the concentrations of t-Hcy, MMA, and cobalamin; Cob, cobalamin, Def, deficiency; IQR, interquartile range; MMA, methylmalonic acid; t-Hcy, total homocysteine.

Values are presented at medians and IQRs. The following cutoff values were used for calculating the percentage with low status of the biomarkers: Cob: <148 pmol/L, t-Hcy: >6.5 μmol/L, MMA: >0.26 μmol/L, and cB12: <-0.5.

Infant plasma cobalamin concentrations at 3 mo of age were estimated to be 33% (9%–63%) higher if the mother used B-vitamin supplements at GW 18 (*P* = 0.005), and 20%(1%–44%) higher at 6 mo of age if the mother used B-vitamin supplements at GW 36. Further, infant t-Hcy concentrations at 6 mo of age were estimated to be 15% (1%–27%) lower if the mother used B-vitamin supplements at GW 18. No associations between maternal B-vitamin supplement use and infant folate status were observed (Table 7).

**TABLE 7:** Infants vitamin B12 and folate status according to maternal supplement use^3^

	Log cob		Log t-Hcy		Log MMA		Log folate		cB12	
	Exp (95%CI)	*P*	Exp (95%CI)	*P*	Exp (95%CI)	*P*	Exp (95%CI)	*P*	Coef. (95%CI)	*P*
Infants’ status 3 mo										
Supplement use GW18, (*n* = 60)^1^										
No	Ref		Ref		Ref		Ref		Ref	
Yes	1.33 (1.09, 1.63)	0.005	0.89 (0.76, 1.05)	0.165	0.95 (0.50, 1.79)	0.863	1.07 (0.81, 1.41)	0.641	0.28 (−0.20, 0.77)	0.249
Supplement use GW36 (*n* = 57)^2^										
No										
Yes	1.10 (0.91, 1.32)	0.323	0.98 (0.84, 1.14)	0.777	1.11 (0.64, 1.91)	0.717	1.00 (0.80, 1.26)	0.985	0.01 (−0.39, 0.42)	0.954
Supplement use 3 mo PP (*n* = 48)										
No	Ref		Ref		Ref		Ref		Ref	
Yes	1.09 (0.91, 1.32)	0.351	0.86 (0.74, 1.00)	0.056	1.00 (0.55, 1.81)	0.995	1.08 (0.84, 1.38)	0.553	0.15 (−0.30, 0.60)	0.512
Infants’ status 6 mo										
Supplement use GW18 (*n* = 59)										
No	Ref		Ref		Ref		Ref		Ref	
Yes	1.21 (0.95, 1.52)	0.118	0.85 (0.73, 0.99)	0.042	0.74 (0.42, 1.28)	0.276	0.88 (0.69, 1.12)	0.295	0.41 (−0.05, 0.86)	0.084
Supplement use GW36 (*n* = 60)										
No	Ref		Ref		Ref		Ref		Ref	
Yes	1.20 (1.01, 1.44)	0.043	0.89 (0.78, 1.02)	0.104	0.91 (0.56, 1.49)	0.707	0.87 (0.71, 1.06)	0.161	0.24 (−0.15, 0.63)	0.223
Supplement use 3 mo PP (*n* = 51)										
No	Ref		Ref		Ref		Ref		Ref	
Yes	1.10 (0.91, 1.33)	0.334	0.92 (0.81, 1.05)	0.212	1.39 (0.85, 2.25)	0.188	1.01 (0.83, 1.24)	0.900	−0.09 (−0.48, 0.29)	0.641
Supplement use 6 mo PP (*n* = 48)										
No	Ref		Ref		Ref		Ref		Ref	
Yes	1.02 (0.82, 1.27)	0.875	0.88 (0.77, 1.02)	0.089	1.26 (0.73, 2.18)	0.398	0.95 (0.77, 1.18)	0.670	−0.06 (−0.48, 0.36)	0.785

Abbreviations: cB12, combined indicator for vitamin B12 status based on the concentrations of t-Hcy, MMA, and cobalamin; CI, confidence interval, cob, cobalamin; GW18, gestation week 18, GW36, gestation week 36; MMA, methylmalonic acid; PP, postpartum; Ref, reference; t-Hcy, total homocysteine.

Generalized linear models were used to investigate the infant’s status at 3 and 6 mo depending on the mothers supplement status at 4 time points (GW18, GW36, and 3 and 6 mo postpartum). Mothers who used multivitamins and/or B-vitamins were considered supplement users while those taking only other supplements or no supplements were considered nonsupplement users. Significant values in bold.

1 *n* = 59 for folate.

2 *n* = 56 for folate.

3 The results are presented as Exp (exponentiated coefficients and corresponding 95% confidence intervals) or Coef (coefficient estimates and corresponding 95% confidence intervals).

## Discussion

In this study of healthy pregnant females and their infants from Norway, we found that a considerable number of infants had low vitamin B12 status at 3 and 6 mo of age, but sufficient folate status. The pregnant females had sufficient vitamin B12 status during pregnancy, but a high proportion had folate deficiency at the end of pregnancy. We also found an association between maternal vitamin B12 status during pregnancy and infant vitamin B12 status at 3 and 6 mo of age. Additionally, the study indicated that breastfed infants had lower vitamin B12 status compared with nonbreastfed infants, and B-vitamin supplement use during pregnancy was associated with better vitamin B12 status among infants during the first 6 mo of life.

In the current study, more than half of the infants had elevated plasma t-Hcy concentrations at 3 and 6 mo. t-Hcy is the preferred marker of vitamin B12 status in the first 2 y of life, but it increases with both vitamin B12 and folate deficiency [16]. In the current study, none of the infants had folate deficiency (defined as plasma folate concentrations <10 nmol/L); thus, the elevated plasma t-Hcy concentrations most likely reflect subclinical vitamin B12 deficiency in this group. Previous studies in Norway have reported a prevalence of approximately 45% to 70% of infants with elevated t-Hcy concentrations [[5], [6], [7]]; thus, our study confirms and adds to these previous findings.

Similar to the high prevalence of subclinical vitamin B12 deficiency, defined as t-Hcy concentrations >6.5 μmol/L in our study, our results suggest the infants also had low vitamin B12 status according to the combined indicator of vitamin B12 status, cB12, where a total of 47% of infants had a low vitamin B12 status at 3 mo, which further increased to 60% at 6 mo. Despite the utility of cB12, taking more than one biomarker of vitamin B12 into account when assessing vitamin B12 status, it is important to note that the use of cB12 as a marker of vitamin B12 status has not yet been validated for use in infancy. Therefore, further studies evaluating the use of the combined indicator in infant populations are necessary. Nonetheless, taking our infant vitamin B12 status observations together with the findings of previous studies suggests that a high proportion of Norwegian infants have biochemical signs of subclinical vitamin B12 deficiency.

Few of the mothers had poor vitamin B12 status during pregnancy, but maternal vitamin B12 status at GW 18 and 36 were moderately associated with infants’ vitamin B12 status at 3 and 6 mo of age. These results are in line with previous studies showing that maternal vitamin B12 status during pregnancy is associated with infant vitamin B12 status from birth until 2 y of age [[32], [33], [34], [35], [36], [37], [38]]. cB12 was the index with the strongest association between the mothers’ and infants’ statuses at all time points. Our results add to the existing knowledge that mothers’ vitamin B12 status during pregnancy is important for infants’ status during the first months of life.

Maternal folate status during pregnancy was not associated with infants’ folate status at 3 and 6 mo of age. Similarly, Hay et al. [33] did not find an association between maternal folate status during pregnancy and the infants’ folate status at 6 mo of age. Our findings indirectly support the view that folate concentration in human milk is relatively stable and independent of the mothers’ folate status [39] and that infants are protected against low folate status even when the mothers’ folate status is low [40].

We found that the t-Hcy in breastfed infants was 44% (4%–100%) higher compared with that of the nonbreastfed infants at age 3 mo, increasing to 55% (30%–85%) higher at age 6 mo. Previous studies among Norwegian and Danish infants also concluded that breastfed infants had lower vitamin B12 status compared with nonbreastfed infants at 6 mo to 2 y of age [41,42]. Also, human milk from well-nourished females contains lower concentrations of vitamin B12 compared with infant formula [43]. In our study, only 3 and 7 infants included in the analysis were not breastfed at 3 and 6 mo, respectively, limiting the strength of our results. However, our results and the abovementioned studies lend further support to the suggestion that breastfed infants may not receive adequate amounts of vitamin B12 throughout the period of recommended breastfeeding.

Overall, maternal B-vitamin supplement use during pregnancy was not clearly associated with vitamin B12 status in infancy. Exceptions were maternal supplement use at GW 18, which was associated with a 33% (9%–63%) increase in infant cobalamin concentrations at 3 mo of age and an 15% (1%–27%) decrease in t-Hcy concentrations at 6 mo of age. Further, supplement use in GW 36 was associated with an 20% (1%–44%) increase in infant cobalamin concentrations at 6 mo of age. Previous studies have shown conflicting results regarding maternal supplement use [33,[44], [45], [46]], where interventions with high-dose vitamin B12 supplements to mothers (≥50 μg/d) have shown the strongest impact on infants’ status [45,46]. Importantly, we did not consider other dietary sources of vitamin B12 and folate, and we grouped all supplement use (multivitamins and/ or B-vitamins) together in our analysis. Thus, more studies are needed to investigate the effect of maternal supplement use on infants’ vitamin B12 and folate statuses.

A strength of our study is the use of both direct and functional biomarkers when assessing vitamin B12 status, as is recommended [18]. We also measured the status in the mothers and infants at 2 time points. However, there was a considerable number of missing data on supplement use among the mothers and biochemical measures of vitamin B12 and folate status in the infants at 3 and 6 mo of age. Furthermore, the mothers in our study had relatively low BMI and high education, which limits the generalizability of our study. However, the participants were recruited through routine ultrasound at the hospital and through online broadcasts, and few infants were born prematurely or with low birth weight. Therefore, our infants are likely a representative sample of healthy Norwegian infants. In our study, we used capillary blood from the fingertip or heel to sample blood when venipuncture was not possible. Sampling capillary blood may result in different concentrations of certain biomarkers, which could have influenced our results.

A major limitation of our study is the cutoff used to define low vitamin B12 status in our participants. There is a lack of consensus regarding the reference values to define deficiency and subclinical deficiency for vitamin B12 and folate, and most of the indicators do not have age-specific cutoffs or cutoffs for pregnant females. Some randomized controlled trials have investigated if supplementing infants with vitamin B12 improves cognitive development [7,10,[47], [48], [49]]. However, the results from these trials are not conclusive, and more studies are needed. Therefore, whether the observed high prevalence of subclinical vitamin B12 deficiency in Norwegian infants reflects a clinical deficiency or if these lower values are normal fluctuations during early life without further consequences is not certain.

In conclusion, we found a high prevalence of subclinical vitamin B12 deficiency in the infants in our study but adequate folate status. Infant’s vitamin B12 status was associated with mother’s status during pregnancy and breastfeeding. Further studies are needed to investigate if this observed subclinical vitamin B12 deficiency has clinical implications for the infants, or if this reflects the need to develop specific cutoffs used to define overt and subclinical vitamin B12 deficiency during infancy.

## Author contributions

The authors’ responsibilities were as follows—MK, LD, MWM: designed and conducted the research; SMGB: analyzed the data, performed the statistical analysis, and wrote the first draft of the paper; CK, TAS, IK: supervised; PMU, AM: analyzed the blood samples; CK, IK, AM, PMU, SNS, LD, MK, TAS, MWM: contributed to the critical revision of the manuscript; and all authors: read and approved the final manuscript.

## Conflict of interest

The authors report no conflicts of interest.

## Funding

The Mommy’s Food study was financially supported by The Norwegian Seafood Research Fund (http://www.fhf.no/) (grant no. 901038) (to MWM). This work was supported by a grant from Innlandet Hospital Trust (grant no. 150455) (to TAS) The funder had no role in the design of the study, the reporting of the results, or the decision to publish this article. The cod used in the trial was purchased, after tender, from Lerøy A/S, Bergen, Norway.

## Data availability

Requests for data collected in the Mommy’s Food study (such as deidentified participant data) can be made to the corresponding author following publication, and requests will be considered on an individual basis. Any requests require completion and approval of the application for use of data from the Mommy's Food study. The trial project group will review and, if acceptable and approved by the Regional Committee for Medical and Health Research Ethics West, Norway, provide approval of the request. A signed data sharing access agreement will be required. To facilitate the data access process, please contact, data will be available after publication of the study results, Maria Wik Markhus at maria.wik.markhus@hi.no and mammasmat@hi.no.
